# Molecular Characterization of Highly Pathogenic Avian Influenza H5N1 Viruses Circulating in Bulgaria During 2024–2025: Evidence for Hidden Circulation and Zoonotic Risk Markers

**DOI:** 10.3390/ijms27041711

**Published:** 2026-02-10

**Authors:** Gabriela Goujgoulova, Georgi Stoimenov, Koycho Koev

**Affiliations:** 1 Risk Assessment Center on Food Chain, 1000 Sofia, Bulgaria; 2 Department of Infectious Pathology and Food Hygiene, Faculty of Veterinary Medicine, University of Forestry, 1796 Sofia, Bulgaria; georgi.stoimenov.vm@gmail.com; 3 Department of Veterinary Microbiology, Infectious and Parasitic Diseases, Faculty of Veterinary Medicine, Trakia University, 6000 Stara Zagora, Bulgaria; koycho.koev@trakia-uni.bg

**Keywords:** avian influenza, Bulgaria, zoonotic markers

## Abstract

The highly pathogenic avian influenza virus HPAI A(H5N1) genotype AF was detected in southern Europe during the 2021/2022 season and spread widely. It emerged in Bulgaria in 2022/2023, mainly affecting mallard ducks. The DA genotype of the virus was detected in a diverse group of birds, including wild birds, zoo birds, and domestic poultry, across a wide area of eastern and southern Europe in 2023. In Bulgaria, following its introduction in 2023, the DA genotype became the predominant virus in laying hens. During 2024–2025, DA spread throughout the country, displacing AF from mallard flocks. The predominant subtype in Europe in 2025 was H5N1 genotype DI.2. This genotype became dominant after December 2024, accounting for over 90% of viruses within the EA-2024-DI genotype lineage, and has been detected in a wide range of bird species. In Bulgaria, DI.2 was identified in only one outbreak in a flock of laying hens in autumn 2024 and in a single case involving a western marsh harrier (*Circus aeruginosus*) in early 2025. These observations are consistent with a pattern of putative hidden circulation of avian influenza virus in duck farms in Bulgaria, potentially establishing a cycle of continuous circulation of the same viral subtype. In this study, we analysed viruses originating from Bulgaria, with a particular focus on EA-2024-DI genotype DI.2, and examined mutations related to host cell receptor binding, host specificity shifts, ligand binding, antibody recognition sites, viral oligomerization interfaces, and other functional regions. Some of these mutations have been associated with antigenic drift, immune escape, and virulence. Importantly, several are linked to changes in host specificity, a critical step in the potential transition of avian influenza viruses to humans. Consequently, such mutations represent key factors in the spread of highly pathogenic avian influenza and may pose a pandemic risk.

## 1. Introduction

Avian influenza viruses (AIVs) represent a persistent and evolving threat to global public health owing to their extensive genetic diversity, broad host range, and capacity for interspecies transmission. Multiple influenza A subtypes circulating in avian reservoirs have caused sporadic zoonotic infections, occasionally resulting in severe disease in humans. Historically, several of the most consequential influenza pandemics have arisen following the successful adaptation of avian-origin viruses to human hosts, underscoring the importance of defining the molecular determinants that govern host specificity, transmissibility, and pathogenicity. Host adaptation of AIVs is a complex, multigenic process that involves coordinated changes in surface glycoproteins, polymerase subunits, and internal structural proteins. Although specific mutations, such as substitutions in the hemagglutinin (HA) receptor-binding site or the polymerase subunit PB2—have been linked to enhanced replication in mammalian hosts, no single genetic marker reliably predicts pandemic potential. Rather, accumulating evidence supports a polygenic model in which incremental and context-dependent mutations collectively shape viral fitness, immune evasion, and host range. This complexity poses a significant challenge for risk assessment frameworks that rely on individual genetic signatures.

Highly pathogenic avian influenza (HPAI) H5N1 was first identified in Bulgaria in 2006 [1]. The avian influenza viruses (AIVs) belonged to clade 2.2. Phylogenetic analysis demonstrated a close relationship between Bulgarian isolates and strains circulating in Europe, the Middle East, and Africa. In the spring of 2010, a new HPAI H5N1 clade 2.3.2.1c outbreak occurred in the Varna region. The virus was detected in a single wild bird (a buzzard) and did not reappear until 2015 [2]. During the winter of 2015, HPAI H5N1 clade 2.3.2.1c was reintroduced into Bulgaria, initially affecting pelicans in the Poda Nature Conservation Center (Burgas region), and, shortly thereafter, a chicken farm in the same area. Subsequently, the virus was detected in other wild and synanthropic birds in this region, as well as in pelicans in the Srebarna Reserve (Silistra region) [3,4].

In December 2016, HPAI H5N8 was introduced into Bulgaria for the first time. Following the initial outbreak, the virus spread rapidly across multiple administrative regions. Between October 2017 and April 2018, 38 outbreaks of HPAI H5 were reported in four distribution zones: Plovdiv and Haskovo in Central Bulgaria, Yambol in Eastern Bulgaria, Dobrich in North-Eastern Bulgaria, and Vidin and Lovech in North-Western Bulgaria. Various bird species were affected, including ducks, chickens, turkeys, partridges, and poultry, both in backyard and commercial farms. Outbreaks in ducks occurred mainly in regions with a high density of these birds. Phylogenetic analysis indicated that Bulgarian H5N8 viruses originated from European H5N8 strains of the 2.3.4.4b lineage.

Genetic analyses suggest that the virus was likely transmitted to poultry by domestic ducks and may have become endemic within the poultry sector. Sequencing of the matrix protein revealed relationships with isolates from multiple species (duck, swan, pheasant) in different countries, including the Netherlands, England, and Hungary [5]. Between March 2019 and February 2021, HPAI H5N8 clade 2.3.4.4b re-emerged in Bulgaria, accompanied by the emergence of a new subtype, H5N2, likely resulting from a local reassortment event. The spread and maintenance of HPAI H5N8 were primarily associated with domestic duck farms, particularly those producing foie gras. Ducks often remain asymptomatic, complicating detection, and rapid production cycles, high movement of birds and personnel, and insufficient disinfection practices contributed to viral persistence. Genetic analyses revealed regional virus movement between farms while maintaining largely localized circulation patterns [6].

In November 2021, H5N1 reappeared and has since remained the dominant strain in the country. Between 2022 and 2023, more than 30 outbreaks were identified across multiple regions (Burgas, Veliko Tarnovo, Gabrovo, Dobrich, Pazardzhik, Pleven, Plovdiv, St. Zagora, Sofia, Haskovo), as well as in one wild bird in Burgas. Genomic characterization of these H5N1 isolates showed that they belonged to clade 2.3.4.4b, with three main circulating genotypes: AF, AP, and DA. Viruses detected in November–December 2021 and in April 2022 and March 2023 belonged to the AF genotype. An outbreak in backyard chickens in Feldfebel Denkovo (Dobrich region) in January 2022 belonged to AP genotype, while isolates from a quail farm in Etropole in January 2023 and viruses collected in October–November 2023 belonged to DA genotype. The DA genotype, also detected in other European countries since September 2023, became predominant in laying hens in Bulgaria [7].

During the 2024–2025 epidemiological year, most HPAI outbreaks in Europe (approximately 90%) were associated with the EA-2024-DI genotype, with DI.2 accounting for about 90% of EA-2024-DI viruses. The detection of older genotypes (EA-2023-DA and EA-2020-AF) in Bulgarian poultry between October 2024 and May 2025, in the absence of detection in wild birds, suggests persistent circulation in unidentified ecological or production niches. In October 2024, DI.2 was detected for the first time in Bulgaria in a large laying-hen farm in Bolyarsko (Yambol region) and was subsequently identified in a wild bird (*Circus aeruginosus*) in early 2025 [8].

The aim of the present study was to analyse avian influenza viruses identified in Bulgaria during 2024–2025 in order to identify key mutations with implications for receptor binding, host specificity shifts, and immune evasion. The region’s dense poultry industry and location along major wild bird migration routes create conditions favourable for the emergence of novel viral variants. These genetic changes highlight the continuous evolutionary potential of avian influenza viruses, which pose ongoing risks for zoonotic spillover and potential pandemics.

Continuous genomic surveillance has therefore become a cornerstone of early warning systems for emerging influenza viruses. Regional studies are particularly valuable, as local ecological conditions, host species composition, and viral lineages can influence evolutionary trajectories. However, data from specific geographical regions remain limited, and the molecular features of circulating influenza viruses are often underrepresented in global datasets. In this regard, South-Eastern Europe occupies a strategically important position along major bird migration routes, facilitating viral exchange between Asia, Europe, and Africa.

In the present study, we performed a comprehensive molecular analysis of avian influenza viruses isolated in Bulgaria over a two-year period. By systematically examining amino acid substitutions in surface glycoproteins, polymerase subunits, and internal proteins, we aimed to identify patterns of antigenic drift, host adaptation, and potential antiviral resistance. Our findings provide region-specific insights into the evolutionary dynamics of avian influenza viruses and contribute to a more integrated understanding of molecular markers relevant to the assessment of zoonotic and pandemic risk.

## 2. Results

**Figure 1:**
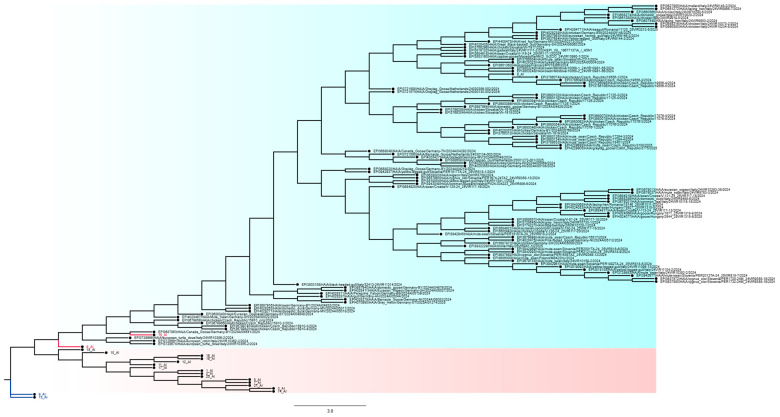
Phylogenetic tree of avian influenza viruses isolated in Bulgaria during 2024–2025. Maximum-likelihood phylogenetic tree of HA sequences. Branch lengths correspond to nucleotide substitutions per site. Bulgarian isolates are highlighted in color, with reference sequences from Italy, Germany, the Netherlands, the Czech Republic, and other European countries included for comparison. Colors indicate clades or subtypes: isolates 13_AI and 9_AI (AF subtype) are colored blue, DA subtype isolates are highlighted in pink, DI subtype isolates are shown in bright green, and Bulgarian isolates 6_AI and 15_AI are marked in red.

We analysed 21 viruses collected through the national monitoring program in Bulgaria during the period 2024–2025. Amino acid substitutions were identified in all viral proteins, as detailed in Supplementary Table S1. Phylogenetic trees were combined with mutation heat maps to facilitate visualization of genetic relationships and substitution patterns (Figure 1). Functional categories shown are based on predictive annotations and previously published studies across multiple influenza A virus subtypes (see Section 4 and Table S1).

### 2.1. Hemagglutinin (HA)

**Figure 2:**
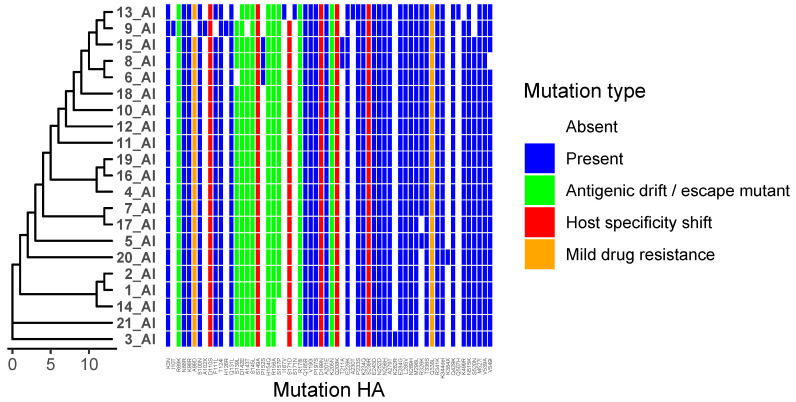
Distribution of Hemagglutinin (HA) Mutations Across Viral Isolates. Heatmap showing the presence and type of HA substitutions in 21 viral isolates (1_AI–21_AI). Rows represent isolates, and columns represent specific HA mutations. Hierarchical clustering of isolates based on mutation profiles is shown on the left. Colours indicate mutation type: white = absent, blue = present, green = antigenic drift/escape mutant, red = host specificity shift, and orange = mild drug-resistance–associated. The figure highlights isolates carrying host specificity shift and/or antigenic drift mutations, as well as clustering patterns reflecting shared mutational profiles.

In the HA protein, multiple amino acid substitutions were detected that cluster in the HA head region, near known receptor-binding and antigenic sites. The most frequently observed substitutions include A143T, S145L, S149A, and K205N (Figure 2). These positions correspond to regions previously described as antigenically relevant in H5 hemagglutinin proteins (see Table S1).

### 2.2. Neuraminidase (NA)

**Figure 3:**
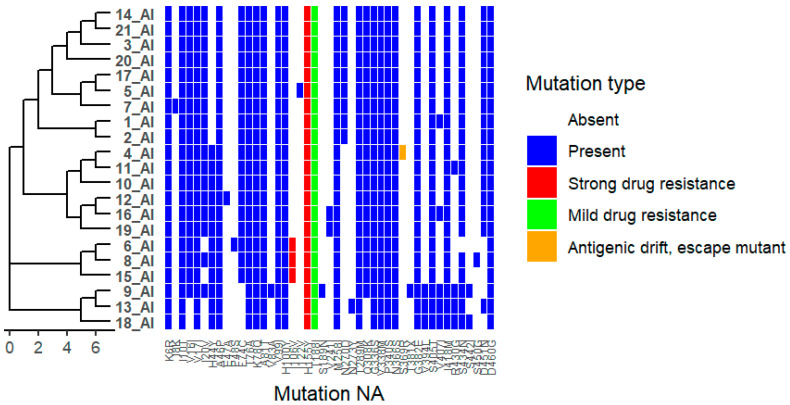
Distribution of Neuraminidase (NA) Mutations Across Viral Isolates. Heatmap showing the presence and type of NA substitutions in 21 viral isolates (1_AI–21_AI). Rows represent isolates, and columns represent specific NA mutations. Hierarchical clustering of isolates based on mutation profiles is shown on the left. Colours indicate mutation type: white = absent, blue = present, red = strong drug-resistance–associated, green = mild drug-resistance–associated, and orange = antigenic drift/escape mutant. The figure highlights isolates carrying drug-resistance substitutions and/or antigenic drift mutations, as well as clustering patterns reflecting shared mutational profiles.

In the neuraminidase (NA) protein, multiple amino acid substitutions were detected across the analysed isolates. Frequently observed substitutions include F74C, T76A, K78Q, V99I, H100Y, T289M, Q308K, G336S, V338M, P340S, N366S, G382E, R430G, S434N, and D460G (Figure 3). These substitutions are located in regions previously described as involved in NA oligomerization or small-ligand–interacting domains. Additional substitutions, including H155Y, I106V, T188I, and S369R, were detected in a limited number of isolates. The functional relevance of these positions has been reported in earlier studies across influenza A virus subtypes (see Table S1).

### 2.3. Polymerase Basic Protein 2 (PB2)

**Figure 4:**
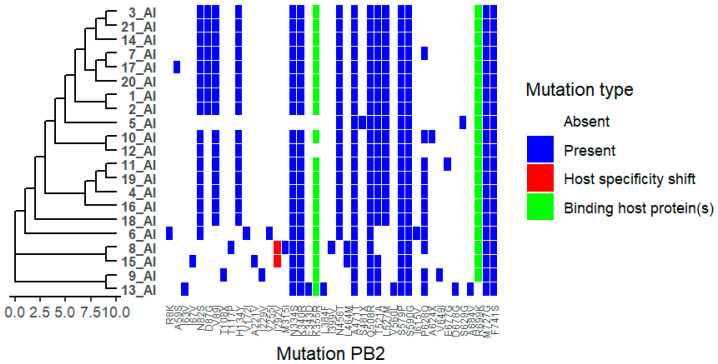
Distribution of Polymerase basic protein 2 (PB2) Mutations Across Viral Isolates. Heatmap showing the presence and type of HA substitutions in 21 viral isolates (1_AI–21_AI). Rows represent isolates, and columns represent specific NA mutations. Hierarchical clustering of isolates based on mutation profiles is shown on the left. Colours indicate mutation type: white = absent, blue = present, red = host specificity shift, green = binding host protein(s). The figure highlights isolates carrying host specificity shift and/or antigenic drift mutations, as well as clustering patterns reflecting shared mutational profiles.

Analysis of the PB2 segment revealed several amino acid substitutions among the Bulgarian isolates. The substitution I292V was identified in isolates 8_AI and 15_AI, while other substitutions, including K355R, R699K, and F741S, were detected across multiple viruses (Figure 4). These substitutions map to regions previously reported to be involved in polymerase activity and interactions with host factors. No classical mammalian-adaptive markers such as E627K or D701N were observed. Functional associations of the detected PB2 substitutions have been described in previous studies (see Table S1).

### 2.4. Polymerase Basic Protein 1 (PB1)

**Figure 5:**
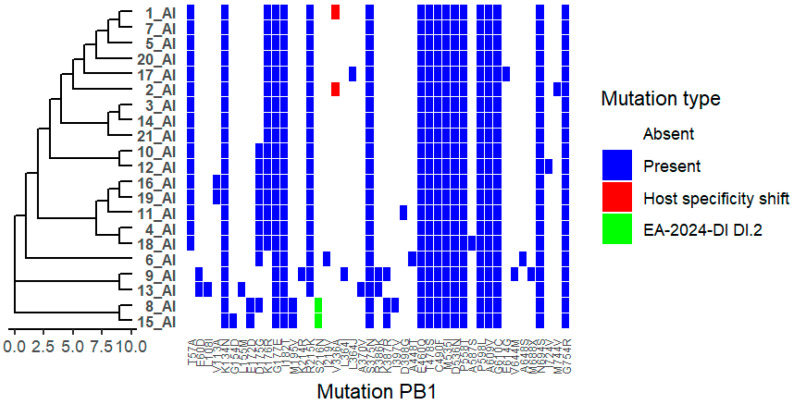
Distribution of Polymerase basic protein 1 (PB1) Mutations Across Viral Isolates. Heatmap showing the presence and type of HA substitutions in 21 viral isolates (1_AI–21_AI). Rows represent isolates, and columns represent specific NA mutations. Hierarchical clustering of isolates based on mutation profiles is shown on the left. Colours indicate mutation type: white = absent, blue = present, red = host specificity shift, green = EA-2024-DI DI.2. The figure highlights isolates carrying host specificity shift and/or antigenic drift mutations, as well as clustering patterns reflecting shared mutational profiles.

In the PB1 protein, a substitution at position 336 was identified in isolates 12_AI and 15_AI (Figure 5). In addition, the substitution S216N was detected in isolates belonging to the DI.2 subtype (8_AI and 15_AI). These positions correspond to regions previously implicated in polymerase function and host adaptation in influenza A viruses. Reported functional associations for PB1 substitutions at these sites are summarized in Table S1.

### 2.5. Polymerase Acidic Protein (PA)

**Figure 6:**
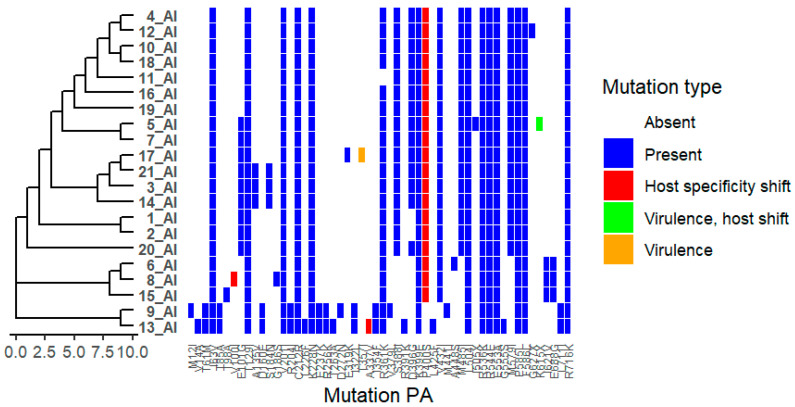
Distribution of Polymerase acidic protein (PA) Mutations Across Viral Isolates. Heatmap showing the presence and type of HA substitutions in 21 viral isolates (1_AI–21_AI). Rows represent isolates, and columns represent specific NA mutations. Hierarchical clustering of isolates based on mutation profiles is shown on the left. Colours indicate mutation type: white = absent, blue = present, red = host specificity shift, green = virulence, host shift, orange = virulence. The figure highlights isolates carrying host specificity shift and/or antigenic drift mutations, as well as clustering patterns reflecting shared mutational profiles.

Multiple substitutions were detected in the PA protein, including V14A, T61M, I63V, T85A, T129I, A135V, D160E, S184N, G186S, I621V, and K615X (Figure 6). Additional substitutions identified include L226F, K228N, A337V, P400S, T357I, and V100I. These residues are located in regions previously described as involved in polymerase assembly, ligand binding, oligomerization interfaces, or host interaction domains. Functional annotations for these substitutions are based on predictive analyses and published studies across influenza A virus subtypes (see Table S1).

### 2.6. Nucleoprotein (NP)

**Figure 7:**
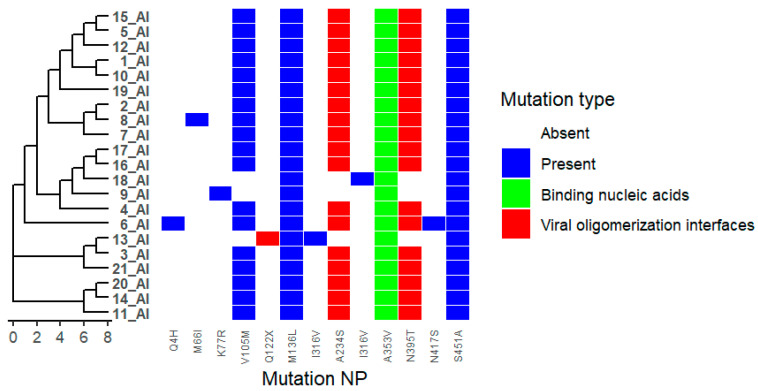
Distribution of Nucleoprotein (NP) Mutations Across Viral Isolates. Heatmap showing the presence and type of HA substitutions in 21 viral isolates (1_AI–21_AI). Rows represent isolates, and columns represent specific NA mutations. Hierarchical clustering of isolates based on mutation profiles is shown on the left. Colours indicate mutation type: white = absent, blue = present, green = binding nucleic acids, red = viral oligomerization interface. The figure highlights isolates carrying host specificity shift and/or antigenic drift mutations, as well as clustering patterns reflecting shared mutational profiles.

In the nucleoprotein (NP) segment, substitutions A234S, N395T, S451A, and Q122X were identified, mapping to regions associated with NP oligomerization interfaces (Figure 7). The substitution A353V was also detected and is located within a region implicated in RNA binding. These positions have been reported in previous studies to influence nucleocapsid assembly and RNA interaction in influenza viruses (see Table S1).

### 2.7. Nonstructural Protein (NS1)

**Figure 8:**
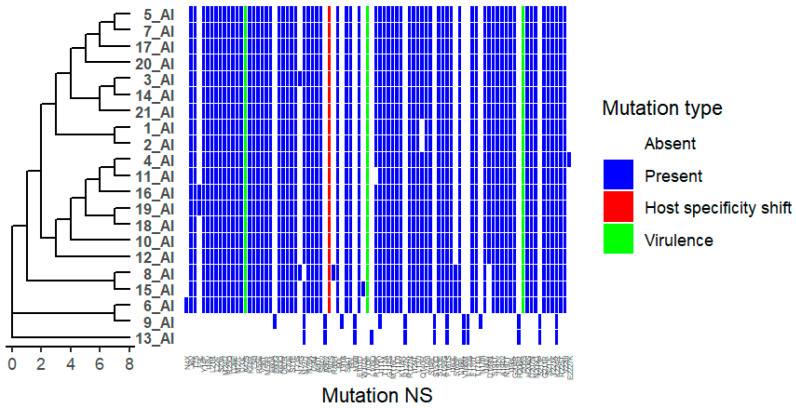
Distribution of Nonstructural protein (NS1) Mutations Across Viral Isolates. Heatmap showing the presence and type of HA substitutions in 21 viral isolates (1_AI–21_AI). Rows represent isolates, and columns represent specific NA mutations. Hierarchical clustering of isolates based on mutation profiles is shown on the left. Colours indicate mutation type: white = absent, blue = present, red = host specificity shift, green = virulence. The figure highlights isolates carrying host specificity shift and/or antigenic drift mutations, as well as clustering patterns reflecting shared mutational profiles.

The NS1 protein exhibited a high number of amino acid substitutions across the analysed viruses. Identified substitutions include I6V, T7S, Y14F, I18V, L21R, L22F, S23A, M24D, R25Q, D26E, M27L, D33L, K44R, L54I, R55E, V56T, M59R, E60A, K63Q, D67R, S71E, T73S, N74D, N76A, I79M, A80T, S84V, I90L, S94T, I95L, E101D, I111V, T112A, G114S, M116C, V117I, K118R, R127N, I129T, L137I, Q140R, V145I, S146L, S153E, F161S, I163L, V166L, F170T, T171D, I180V, D189N, I192V, A194V, N197T, I198L, G204R, and H206S (Figure 8). Additional substitutions, including A42S, Y103F, I205S, K70E, and P87S, were also observed. These substitutions map to regions previously associated with NS1 oligomerization, host protein interactions, immune modulation, or virulence in influenza A viruses (see Table S1).

### 2.8. Matrix Protein (M1)

**Figure 9:**
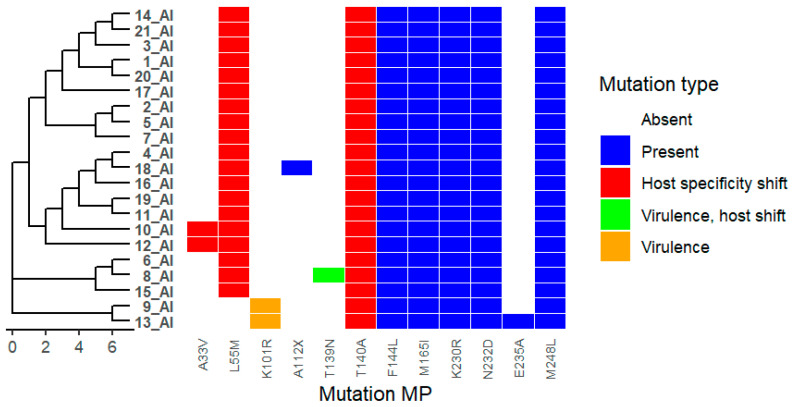
Distribution of Matrix protein (M1) Mutations Across Viral Isolates. Heatmap showing the presence and type of HA substitutions in 21 viral isolates (1_AI–21_AI). Rows represent isolates, and columns represent specific NA mutations. Hierarchical clustering of isolates based on mutation profiles is shown on the left. Colours indicate mutation type: white = absent, blue = present, red = viral oligomerization interfaces, green = virulence, orange = viral oligomerization interfaces, virulence. The figure highlights isolates carrying host specificity shift and/or antigenic drift mutations, as well as clustering patterns reflecting shared mutational profiles.

In the matrix protein M1, the substitution T139N was identified, along with additional substitutions including K101R, A33V, L55M, and T140A (Figure 9). These residues are located in regions previously described as involved in M1 oligomerization interfaces and virion assembly. Functional associations reported for these positions in influenza A viruses are summarized in Table S1.

## 3. Discussion

The largest pandemics in human history have often resulted from the adaptation of AIVs to human hosts. The precise amino acid mutations required for AIVs to efficiently replicate in humans remain incompletely defined. Finkelstein et al. (2007) performed extensive sequence analyses and statistical testing across influenza virus sequences and identified 32 molecular markers distinguishing human influenza viruses from avian viruses [9]. Their findings indicated that H5N1 viruses circulating today are no more adapted to humans than in previous years. Nevertheless, AIVs continue to pose a substantial pandemic risk, particularly if they acquire additional mutations that facilitate human infection or increase pathogenicity [10]. Here, we summarize and analyse molecular markers identified in avian influenza viruses isolated in Bulgaria over the past two years.

In this study, the term “hidden circulation” refers to the inferred persistence of avian influenza virus lineages within domestic or semi-domestic avian populations that are not readily detected through routine surveillance of wild birds. Evidence supporting this interpretation includes the repeated detection of older genotypes (EA-2020-AF and EA-2023-DA) in Bulgarian poultry during 2024–2025, despite their near disappearance from wild bird surveillance data at the European level. The continued identification of these genotypes, particularly in domestic ducks and poultry farms, suggests localized maintenance in production systems or ecological niches with limited outward detection rather than repeated re-introduction from migratory wild birds. While direct transmission pathways cannot be confirmed from genomic data alone, the observed temporal persistence and host association patterns are consistent with ongoing, low-visibility circulation within domestic bird populations.

The combination of A143T, S145L, and S149A in HA has been reported as a pattern associated with antigenic masking and altered antibody binding [11]. Host adaptation, such as increased α2,6 sialic acid receptor binding or enhanced polymerase activity, typically requires additional mutations, for example in the receptor-binding site (RBS) core residues or PB2 (E627K, D701N) [12]. HA substitutions such as D142E and S145L are frequently observed in public databases, suggesting that they may be clade-associated or established mutations. Less common variants, including K205N or A143T in specific contexts, may be of greater interest when they occur alongside these more prevalent substitutions [13].

Many NA substitutions, including F74C, T76A, K78Q, V99I, H100Y, T289M, Q308K, G336S, V338M, P340S, N366S, G382E, R430G, S434N, and D460G, are located in regions involved in NA oligomerization or small-ligand binding. Changes in these regions may influence NA structural stability or interactions with substrates or inhibitors, even if they do not directly affect the active site [13]. Substitutions such as H155Y and I106V have been documented in multiple influenza systems as part of mutation combinations associated with reduced neuraminidase inhibitor (NAI) effectiveness [14]. T188I has been associated with moderate reductions in antiviral susceptibility and may also influence antibody recognition of NA, potentially impacting antigenicity [15]. The substitution S369R, observed in isolate 4_AI, is consistent with NA mutations that contribute to antigenic drift and immune evasion, underscoring the role of NA in influenza evolution and vaccine effectiveness [16].

PB2 is a critical subunit of the influenza A RNA polymerase, responsible for “cap snatching” and interactions with host factors that influence replication efficiency and host range [17]. Major mammalian-adaptive mutations, such as PB2 E627K or D701N, were not observed in the Bulgarian isolates analysed in this study [18]. However, minor or rare mutations can modulate polymerase function when combined with other adaptive changes [19]. For example, PB2 I292V, detected in isolates 8_AI and 15_AI, has been reported to enhance replication efficiency and innate immune evasion in mammalian hosts, although it does not act as a standalone virulence determinant. This mutation has become prevalent in several avian H9N2 lineages over the past two decades and has also been detected in pandemic H1N1 and human-infecting H7N9 and H10N8 viruses, suggesting positive selection at the bird–mammal interface [20].

PA mutations such as P400S, K228N, I63V, and T129I were frequently observed and may reflect adaptive significance related to polymerase function or host interactions. Less common substitutions, including A337V, T357I, and V100I may influence virulence or host adaptation when present in combination with other mutations [21,22]. Structural analyses indicate that several of these residues (I63V, T129I, V100I) are located within oligomerization interfaces or ligand-binding sites, emphasizing their potential impact on polymerase assembly and viral replication efficiency [23]. Experimentally, V100I in combination with other PA mutations has been shown to enhance replication and transmission in mammalian models [24].

Mutations at NP oligomerization interfaces including A234S, N395T and S451A, may influence assembly efficiency [25,26,27]. Substitutions at RNA-binding sites, such as A353V, directly affect NP-RNA interactions. The high prevalence of A353V and S451A suggests positive selection, whereas A234S and N395T appear less common and may be context-dependent [28].

NS1 and NS2 substitutions including A42S, P87S, I205S, and NS2 N67E, have been associated with altered NS protein functionality, virulence, and host adaptation [29,30]. NS1 substitutions such as K70E and P87S have been linked to shifts in host range [31,32]. The rare NS2 L13M substitution appears strain-specific. Many NS mutations occur at oligomerization interfaces or host protein-binding regions, highlighting the central role of NS proteins in viral replication and immune modulation [29,33].

Mutations in matrix proteins further emphasize the functional importance of structural interfaces in viral assembly and pathogenicity. In particular, M1 substitutions tend to cluster at oligomerization interfaces and may influence virion morphology, stability, and virulence [34]. The T139N substitution has been linked to altered virulence in specific genetic backgrounds [35]. In addition, substitutions in the M2 proton channel, such as V27A, confer resistance to adamantane antivirals, including amantadine, underscoring the continued relevance of resistance monitoring even for legacy drug classes [36].

Collectively, the molecular profile of avian influenza viruses isolated in Bulgaria reflects ongoing antigenic drift and gradual host adaptation rather than the emergence of fully human-adapted strains. The observed constellation of mutations supports a polygenic model in which pandemic potential arises through the accumulation and interaction of multiple substitutions with modest individual effects across viral proteins. These findings reinforce the importance of continuous genomic surveillance and integrative, multigenic analyses to improve early risk assessment of zoonotic influenza viruses and to inform preparedness strategies.

## 4. Materials and Methods

### 4.1. Viruses

In this study, we analysed 21 avian influenza viruses (AIVs) collected in Bulgaria during the period 2024–2025 (Table 1). All viral data were officially recorded in the Animal Disease Information System (ADIS). Samples were collected as part of the national avian influenza surveillance program and initially tested at the National Reference Laboratory for Avian Influenza (Sofia, Bulgaria). The author team was responsible for epidemiological data interpretation, sequence analysis, phylogenetic reconstruction, mutation annotation, and functional interpretation of the viral genomes, while sequencing and primary sequence submission to GISAID [37] were performed by the Istituto Zooprofilattico Sperimentale delle Venezie, Italy.

### 4.2. Phylogenetic Analyses

To investigate the genetic relationships of the Bulgarian isolates, reference sequences were downloaded from the GISAID database [9]. Sequences were manually curated and aligned using MEGA 11.0.13. Phylogenetic trees were reconstructed using the maximum likelihood (ML) method with the Tamura–Nei model to estimate evolutionary distances and relationships. The graphical representation of phylogenetic trees was generated using FigTree v1.4.4 [38]. For further computational analyses, trees were saved in Newick format and imported into RStudio for additional processing, visualization, and annotation.

### 4.3. FluServer Mutation Tool

To assess specific molecular markers, viral sequences were analyzed using the FluSurver mutation tool (http://flusurver.bii.a-star.edu.sg) [28]. Each Bulgarian isolate was compared with the reference strain A/Goose/Guangdong/1/1996(H5N1). The analysis focused on mutations affecting host receptor specificity, interactions with host proteins, viral oligomerization interfaces, ligand-binding sites, and other functional regions known to influence viral replication, host adaptation, and immune evasion. This approach allowed the identification of mutations of potential functional significance in the context of host adaptation and pathogenicity.

Functional annotations assigned to amino acid substitutions (e.g., antigenic drift, host specificity shift, immune escape, or drug resistance) are based on in silico predictions generated using the FluSurver mutation analysis tool and on previously published studies across multiple influenza A virus subtypes and host systems. These functional labels reflect reported or predicted effects under specific experimental or epidemiological contexts and do not represent direct experimental validation for the H5N1 clades analyzed in this study. Accordingly, functional interpretations should be regarded as indicative rather than definitive for the Bulgarian H5N1 viruses.

### 4.4. Data Management and Statistical Analysis

All data obtained from the active AIV surveillance program were compiled in CSV (comma-separated values) files for standardized storage and processing. Statistical analyses and data visualization were performed using RStudio version 2025.09.2+418 (RStudio Inc., Boston, MA, USA). This included descriptive statistics, frequency analyses of detected mutations, and integration of phylogenetic data with mutation profiles. Graphical outputs were generated to summarize mutational patterns and highlight trends in the evolution of H5 viruses circulating in Bulgaria.

## 5. Conclusions

Analysis of avian influenza viruses isolated in Bulgaria during 2024–2025 reveals a complex constellation of molecular markers influencing host adaptation, antigenicity, and antiviral susceptibility. While major mammalian-adaptive mutations such as PB2 E627K and D701N were absent, several minor or rare substitutions—particularly PB2 I292V, PA V100I, and HA antigenic site changes (A143T, S145L, S149A)—may modulate viral replication and immune evasion in mammalian hosts. Frequent HA and NA substitutions, including D142E, S145L, and S369R, indicate ongoing antigenic drift with potential implications for vaccine efficacy.

Mutations in polymerase subunits (PB2, PA) and NP suggest adaptive changes that could subtly enhance replication efficiency, host range, or virulence, particularly in combination with other substitutions. NS1 and NS2 mutations appear to fine-tune host immune modulation, while M1 and M2 interface substitutions affect viral assembly, morphology, and antiviral resistance. Collectively, these findings indicate that although Bulgarian AIVs currently lack a full complement of human-adaptive changes, they harbour multiple substitutions that may incrementally increase pandemic potential through further adaptation.

Overall, this study provides a comprehensive molecular characterization of avian influenza viruses circulating in Bulgaria and supports a polygenic model of host adaptation driven by the accumulation and interaction of modest-effect mutations across viral proteins. These results underscore the importance of continuous genomic surveillance and integrative, multi-gene analyses to improve early risk assessment, guide vaccine design and antiviral strategies, and strengthen preparedness at the bird–human interface.

## Figures and Tables

**Table 1:** Avian influenza virus samples collected in Bulgaria (2024–2025).

Code	Samples	Accession Number	Subtype	Administrative Division Level 1	Administrative Division Level 2
1_AI	A/laying-hen/Bulgaria/679AI_25VIR7160-9/2025	EPI_ISL_20141170	H5N1	Burgas	Aitos
2_AI	A/laying-hen/Bulgaria/355AI_25VIR7160-8/2025	EPI_ISL_20141169	H5N1	Plovdiv	Rodopi
3_AI	A/domestic-duck/Bulgaria/247AI_25VIR7160-7/2025	EPI_ISL_20141168	H5N1	Plovdiv	Rakovski
4_AI	A/duck/Bulgaria/274_24VIR2991-16/2024	EPI_ISL_19132711	H5N1	Dobrich	Dobrich
5_AI	A/pheasant/Bulgaria/1543_24VIR11025-14/2024	EPI_ISL_19701032	H5N1	Plovdiv	Rakovski
6_AI	A/duck/Bulgaria/243_24VIR2991-14/2024	EPI_ISL_19132710	H5N1	Veliko Tarnovo	Gorna Oryahovitsa
7_AI	A/laying-hen/Bulgaria/1619_24VIR11025-15/2024	EPI_ISL_19701033	H5N1	Plovdiv	Plovdiv
8_AI	A/laying-hen/Bulgaria/1654_24VIR11025-16/2024	EPI_ISL_19701034	H5N1	Yambol	Tundzha
9_AI	A/duck/Bulgaria/48_24VIR2991-5/2024	EPI_ISL_19132709	H5N1	Plovdiv	Bresovo
10_AI	A/laying-hen/Bulgaria/607_24VIR11025-5/2024	EPI_ISL_19701028	H5N1	Kardzhali	Krumovgrad
11_AI	A/broiler/Bulgaria/631_24VIR11025-6/2024	EPI_ISL_19701029	H5N1	Haskovo	Haskovo
12_AI	A/laying-hen/Bulgaria/702_24VIR11025-7/2024	EPI_ISL_19701030	H5N1	Plovdiv	Asenovgrad
13_AI	A/domestic-duck/Bulgaria/860_24VIR11025-10/2024	EPI_ISL_19701031	H5N1	Haskovo	Simeonovgrad
14_AI	A/domestic-duck/Bulgaria/173AI_25VIR7160-4/2025	EPI_ISL_20141151	H5N1	Plovdiv	Maritsa
15_AI	A/western-marsh-harrier/Bulgaria/118AI_25VIR7160-3/2025	EPI_ISL_20141150	H5N1	Burgas	Burgas
16_AI	A/hens/Bulgaria/517_24VIR2991-31/2024	EPI_ISL_19132750	H5N1	Plovdiv	Rodopi
17_AI	A/laying-hen/Bulgaria/107AI_25VIR7160-2/2025	EPI_ISL_20141149	H5N1	Plovdiv	Asenovgrad
18_AI	A/hens/Bulgaria/309_24VIR2991-20/2024	EPI_ISL_19132749	H5N1	Haskovo	Dimitrovgrad
19_AI	A/laying-hen/Bulgaria/555_24VIR11025-2/2024	EPI_ISL_19701027	H5N1	Haskovo	Haskovo
20_AI	A/domestic-duck/Bulgaria/195AI_25VIR7160-5/2025	EPI_ISL_20141157	H5N1	Plovdiv	Asenovgrad
21_AI	A/domestic-duck/Bulgaria/224AI_25VIR7160-6/2025	EPI_ISL_20141167	H5N1	Plovdiv	Rakovski

## Data Availability

FluSurver Database, mutation prevalence and functional annotations. https://flusurver.bii.a-star.edu.sg (accessed 12 November 2025); Global Initiative on Sharing All Influenza Data. Available online: https://gisaid.org/ (accessed 12 November 2025).

## Supplementary Materials

The following supporting information can be downloaded at: https://www.mdpi.com/article/10.3390/ijms27041711/s1, References [39,40,41,42,43,44,45,46,47,48,49] are cited in the supplementary materials.

## Abbreviations

The following abbreviations are used in this manuscript:

ADIS	Animal Disease Information System
GISAID	Global Initiative on Sharing All Influenza Data
CDC	Centers for Disease Control and Prevention
