# Supplementary material for: Molecular Characterization of Highly Pathogenic Avian Influenza H5N1 Viruses Circulating in Bulgaria During 2024–2025: Evidence for Hidden Circulation and Zoonotic Risk Markers
Source: Int J Mol Sci. 2026 Feb 10;27(4):1711. doi: 10.3390/ijms27041711 (PMC12940615; doi:10.3390/ijms27041711)
Supplement: Supplementary file 1 [file ijms-27-01711-s001.zip › ijms-4116240-supplementary.docx]

Supplemental table S1. Grouped Mutation Table with Literature Citations

| Gene | Mutation Group | Function | Literature |
| --- | --- | --- | --- |
| HA | K3N, I10T, Q429K, N515K, S520N, V549I, A279T | Viral oligomerization interfaces | [28] |
| HA | R69K | Antigenic drift / escape mutant; viral oligomerization interfaces; binding small ligand(s) | [11] |
| HA | N88R, K98R, S100N, Q131L, S139L, A201E, E228K, N256H, K282R, E284G, N289H, Q338L, K499R | Viral oligomerization interfaces; binding small ligand(s); antibody recognition sites | [11] |
| HA | A99D, P233S, L285V, T336S, Q507H | Viral oligomerization interfaces; antibody recognition sites | [11] |
| HA | A102X, Q185R, D199N, E243D, N252D, M298L, R326K | Viral oligomerization interfaces; binding small ligand(s) | [11] |
| HA | D110S | Viral oligomerization interfaces; binding small ligand(s); antibody recognition sites; involved in a T-cell epitope presented by MHC molecules | [39] |
| HA | F111L | Viral oligomerization interfaces; binding small ligand(s); involved in a T-cell epitope presented by MHC molecules | [39] |
| HA | T124I | Viral oligomerization interfaces; involved in a T-cell epitope presented by MHC molecules | [39] |
| HA | H126R | Viral oligomerization interfaces; binding small ligand(s); involved in a T-cell epitope presented by MHC molecules | [39] |
| HA | D142E | Related to virulence; antigenic drift / escape mutant; viral oligomerization interfaces; binding small ligand(s); antibody recognition sites | [11,12] |
| HA | A143T | Host cell receptor binding; antigenic drift / escape mutant; viral oligomerization interfaces; binding small ligand(s); antibody recognition sites | [11,40] |
| HA | S145L | Related to host specificity shift; related to virulence; antigenic drift / escape mutant; viral oligomerization interfaces; binding small ligand(s); antibody recognition sites | [11,12] |
| HA | S149A | Related to host specificity shift; host cell receptor binding; viral oligomerization interfaces; binding small ligand(s); antibody recognition sites | [11] |
| HA | P152S, H154Q, R156A | Antigenic drift / escape mutant; viral oligomerization interfaces; binding small ligand(s); antibody recognition sites | [11] |
| HA | S157P | Related to virulence; antigenic drift / escape mutant; viral oligomerization interfaces; binding small ligand(s); antibody recognition sites | [11] |
| HA | I167V | Related to host specificity shift; host cell receptor binding; antigenic drift / escape mutant; viral oligomerization interfaces; binding small ligand(s); antibody recognition sites | [11] |
| HA | S171D | Related to host specificity shift; viral oligomerization interfaces; binding small ligand(s); antibody recognition sites | [11] |
| HA | R178I | Antigenic drift / escape mutant; viral oligomerization interfaces; binding small ligand(s); antibody recognition sites | [11] |
| HA | K205N | Related to virulence; antigenic drift / escape mutant; host cell receptor binding; viral oligomerization interfaces; binding small ligand(s); antibody recognition sites; involved in a T-cell epitope presented by MHC molecules and binding host protein(s) | [11,40] |
| HA | Q208K | Related to host specificity shift; viral oligomerization interfaces; antibody recognition sites; involved in a T-cell epitope presented by MHC molecules | [11] |
| HA | T211A | Viral oligomerization interfaces; involved in a T-cell epitope presented by MHC molecules | [11] |
| HA | A230T | Related to host specificity shift and virulence; viral oligomerization interfaces | [11] |
| HA | K234Q | Related to virulence and host specificity shift; viral oligomerization interfaces; binding small ligand(s); antibody recognition sites | [11] |
| HA | S239R | Related to host specificity shift; host cell receptor binding; viral oligomerization interfaces; binding small ligand(s); antibody recognition sites | [11] |
| HA | K384X | Viral oligomerization interfaces; binding small ligand(s); involved in binding host protein(s) | [11] |
| HA | P48S, V83A, S189N, N273Y, T289M, T381X, V407I | Binding small ligand(s) | [41] |
| HA | F74C, T76A, K78Q, V99I, Q308K, G336S, V338M, G382E, R430G, V394L, D460G | Viral oligomerization interfaces | [41] |
| HA | H100Y, N270D | Viral oligomerization; ligand binding | [41] |
| HA | I106V | Strong drug resistance; viral oligomerization; ligand binding | [42] |
| HA | H155Y | Strong drug resistance; oligomerization; ligand binding; antibody recognition | [42] |
| HA | T188I | Mild drug resistance; viral oligomerization | [42] |
| HA | P340S, N366S, S434N | Oligomerization; ligand binding; antibody sites | [41] |
| HA | S369R | Antigenic drift / escape; oligomerization; ligand binding; antibody sites | [43] |
| HA | S450G, D451N | Binding host proteins; oligomerization; ligand binding | [43] |
| NA | P48S, V83A, S189N, N273Y, T289M, T381X, V407I | Binding small ligand(s) | [41] |
| NA | F74C, T76A, K78Q, V99I, Q308K, G336S, V338M, G382E, R430G, V394L, D460G | Viral oligomerization interfaces | [41] |
| NA | H100Y, N270D | Viral oligomerization; ligand binding | [41] |
| NA | I106V | Strong drug resistance; viral oligomerization; ligand binding | [42] |
| NA | H155Y | Strong drug resistance; oligomerization; ligand binding; antibody recognition | [42] |
| NA | T188I | Mild drug resistance; viral oligomerization | [42] |
| NA | P340S, N366S, S434N | Oligomerization; ligand binding; antibody sites | [41] |
| NA | S369R | Antigenic drift / escape; oligomerization; ligand binding; antibody sites | [43] |
| NA | S450G, D451N | Binding host proteins; oligomerization; ligand binding | [43] |
| PB 2 | R8K, N334S, K340R, E343D, L384F, L464M, S481X, A684V | Viral oligomerization interfaces | [44] |
| PB2 | I63V, V89I | Virulence | [44] |
| PB2 | I292V | Host specificity shift | [45] |
| PB 2 | K355R, N456T | Oligomerization; ligand binding | [45] |
| PB 2 | V560L | T-cell epitope | [46] |
| PB 2 | S590G, P628Q, A624X, E677G, D678G | Ligand binding | [45] |
| PB2 | R699K | Oligomerization; host protein binding | [44] |
| PB2 | F741S | Host protein binding | [45] |
| PB1 | V336A | Host specificity shift | [46] |
| PB1 | S375N | Virulence | [46] |
| PB1 | P598L | Polymerase activity (enhanced in mammalian cells) | [47] |
| PB1 | M688X, N694S | Ligand binding | [46] |
| PB1 | G754R | Oligomerization; ligand binding | [44] |
| PA | M12I, T98A, A135V, D160E, S184N, G186S | Viral oligomerization | [48] |
| PA | V14A, T61M, I63V, T85A, T129I | Oligomerization; ligand binding | [48] |
| PA | V100I | Oligomerization; host specificity shift | [43] |
| PA | L226F, K228N | T-cell epitope | [48] |
| PA | T357I | Virulence | [43] |
| PA | A337V, P400S | Host specificity shift | [43] |
| PA | K615X | Virulence; host specificity shift | [48] |
| PA | I621V | Ligand binding | [48] |
| NP | Q122X, A234S, N395T, N417S, S451A | Viral oligomerization | [44] |
| NP | A353V | Nucleic acid binding | [44] |
| NS1 | N4X, I6V, S23A, D33L, I90L, W102L, I192V, A194V, N197T | Oligomerization; ligand binding | [43] |
| NS1 | T7S, L22F, M24D, R55E, L21R, R25Q, D26E, M27L, D67R, S71E, T73S, N74G/N74D, R88H, E101D, T112A, V117I, K118R, R127K/R127N, I129T, L137I, V145I, S165F, V166L/M, F170S/T, T171D, N176I | Oligomerization; ligand binding; host binding | [43] |
| NS1 | F9L, Y14F, I18V, K44R, I79M, A80T, S84V, A86V, I111V, M116C, I163L, I198L, G204R, H206R/H206S | Oligomerization | [43] |
| NS1 | A42S | Virulence; oligomerization; ligand binding; nucleic acid binding | [43] |
| NS1 | L54I | Ligand binding | [43] |
| NS1 | V56T, M59R, E60A/G, K63Q, N76A | Oligomerization; ligand binding | [43] |
| NS1 | K70E | Host specificity shift; oligomerization; ligand binding | [43] |
| NS1 | P87S | Host specificity shift; oligomerization; host protein binding | [43] |
| NS1 | T91A, I95L, M98I | Oligomerization; ligand binding; MHC epitope; host binding | [43] |
| NS1 | S94T | Oligomerization; ligand binding; MHC epitope | [43] |
| NS1 | Y103F | Oligomerization; ligand binding; host binding; virulence | [43] |
| NS1 | L105Q, R108K, D189N | Oligomerization; host binding | [43] |
| NS1 | G114S | Host binding | [43] |
| NS1 | Q140R, S146L, S153D/E, F161S, I180V | Oligomerization; ligand binding; host binding | [43] |
| NS1 | I205S | Virulence; oligomerization | [43] |
| NS2 | Q14M, S48A, N67E | Virulence | [43] |
| NS2 | C83V, I86R | Oligomerization | [43] |
| NS2 | L100M | Virulence | [43] |
| M1 | A33V | Oligomerization | [44] |
| M1 | L55M | Oligomerization | [44] |
| M1 | K101R | Virulence; oligomerization | [44] |
| M1 | T139N | Virulence | [44] |
| M1 | T140A | Oligomerization | [44] |
| M2 | D24N, V28I | Oligomerization; ligand binding | [42] |
| M2 | V27A | Strong & mild drug resistance; drug binding; oligomerization; ligand binding | [49] |
| M2 | C50Y, I51V | Oligomerization | [44] |
